# Hepatitis B and C prevalence among hemodialysis patients in the West Bank hospitals, Palestine

**DOI:** 10.1186/s12879-016-1359-8

**Published:** 2016-02-01

**Authors:** Hamzeh Al Zabadi, Hani Rahal, Rasha Fuqaha

**Affiliations:** 1 Public Health Department, Faculty of Medicine and Health Sciences, An-Najah National University, P.O. Box 7, Nablus, West Bank Palestine; 2 Jenin Governmental Hospital, Jenin, West Bank Palestine

**Keywords:** Hemodialysis, Hepatitis B virus, Hepatitis C virus, West Bank, Palestine

## Abstract

**Background:**

Hepatitis B and C virus infection is a lead cause of morbidity and mortality among hemodialysis patients. Yet, little research has focused on the morbidity measures of these serious disorders in low and middle income countries. The study aims to estimate the prevalence of hepatitis B and C among hemodialysis patients in the West Bank hospitals in Palestine.

**Methods:**

A retrospective medical records review design was performed for all governmental and private hospitals in the West Bank which provide hemodialysis services for the patients. Data was retrieved from the patients’ medical files and from the computerized health information system in some hemodialysis centers. SPSS software version 16 was used for data entry and analysis.

**Results:**

In overall, 868 hemodialysis patients attending nine hemodialysis hospitals in the West Bank was recruited. The overall prevalence of hepatitis B virus was found to be 3.8 % (33 cases) with a range from 0.0 % (in Jericho and Qalqelia districts) to 11.8 % (in Bethlehem district). Regarding hepatitis C virus, the overall prevalence was estimated around 7.4 % (64 cases) with a range from 2.9 % (in Nablus district) to 15.9 % (in Qalqelia district).

**Conclusions:**

Although relatively low prevalence of both hepatitis B and C virus was found in a couple of hemodialysis hospitals, some higher prevalence values urge for the implementation of stricter infection prevention measures and more effective follow up procedures.

## Background

The primary purpose of the renal system is to maintain the body’s state of homeostasis by carefully regulating fluid and electrolytes, removing wastes, and providing other functions [1]. Dysfunction of the kidneys is common and may occur at any age and with varying degrees of severity [2]. Chronic kidney disease (CKD) is an umbrella term that describes kidney damage or a decrease in the glomerular filtration rate lasting for three or more months. CKD is associated with decreased quality of life, increased health care expenditures, and premature death [3]. Untreated CKD can result in end-stage kidney disease (ESKD), which is the final stage of renal failure [4]. ESKD results in retention of uremic waste products and the need for renal replacement therapies, dialysis, or kidney transplantation [2]. The cause of renal failure may be a primary kidney disorder or secondary to a systemic disease or other urologic defects. Hemodialysis is used for patients who are acutely ill and require short-term dialysis ranging from days to weeks until kidney resumes its function as well for patients with advanced CKD and ESKD who require long-term or permanent renal replacement therapy [2].

In hemodialysis, blood is removed from the patient with needles and plastic tubing and pumped past the dialysis membrane. Poisons and toxins cross the dialysis membrane into the dialysate, which is then discarded, and the blood is returned to the patient [5]. Viral hepatitis and human immunodeficiency virus infection are lead causes of mortality and morbidity in patients with hemodialysis (HD). Both are further promoted by the characteristic immunological dysfunction that develops in renal failure and interferes with the patient’s ability to eliminate these viruses. As far as HD is concerned, hepatitis B virus (HBV) and hepatitis C virus (HCV) are the two most important viruses responsible for almost all the patients’ morbidity [6]. Table 1 below shows some worldwide prevalence of HBV and HCV among HD patients [7, 8].

**Table 1:** The worldwide prevalence of HBV and HCV among hemodialysis patients (The table was adopted from references 7 and 8)

Country	Year	HBV^a^		HCV^b^	
		Total n° of patients	Percent	Total n° of patients	Percent
Bahrain	2004	81	11.8	NA^c^	NA
Belgian	2000	NA	NA	1710	6.8
Brazil	2006	1095	29.8	NA	NA
Brazil	2002	NA	NA	795	16.5
Brazil	2001	NA	NA	428	39
Germany	2002	NA	NA	2796	6.1
Germany	1992	NA	NA	122	3.3
Greece	2006	49	20.4	NA	NA
Greece	2005	NA	NA	366	24
India	2005	75	14.2	134	5.9
Indonesia	2002	NA	NA	93	63.4
Indonesia	1996	NA	NA	76	76.3
Iran	2005	324	4.6	NA	NA
Iran	2003	NA	NA	838	21.0
Israel	1999	NA	NA	65	24.6
Jordan	2008	427	5.9	NA	NA
Jordan	2002	NA	NA	283	34.6
Kenya	2003	100	8	NA	NA
Lebanon	1996	NA	NA	317	27.0
Mexico	2004	NA	NA	149	6.7
Morocco	2005	186	2	NA	NA
Pakistan	2004	97	12.4	NA	NA
Saudi Arabia	2001	67	10	NA	NA
Spain	2005	86	20.9	NA	NA
Switzerland	2000	1713	1.63	NA	NA
Syria	1998	NA	NA	120	75.0
Tunisia	2001	NA	NA	4340	19.1
Turkey	2006	188	25	NA	NA
USA	2001	252739	0.9	NA	NA
Vietnam^!^	2013	113	7.0	113	6.0
*Co-infection 1 %*					

^a^ *HBV* hepatitis B virus, ^b^ *HCV* hepatitis C virus. ^c^ *NA* not applicable^!^

In Gaza strip in Palestine, a study found that the overall prevalence of HBV and HCV among the HD patients was 8.1 and 22 %; respectively [7]. However, no documented data is available regarding the West Bank. Therefore, this study aims to estimate the prevalence of HBV and HCV among HD patients in the West Bank of Palestine in the period from October to November 2014.

## Methods

### Study design, settings and population

A retrospective medical records review design was conducted. The study population included all patients who undergo HD in the West Bank including all governmental and nongovernmental (An-Najah National University Teaching Hospital) HD centers at the time of study.

### Sample size

A total number of 868 patients were included in the study during the period from October to November 2014.

### Ethical considerations

The study protocol was approved by An-Najah National University IRB (Institutional Review Board) committee. Permissions and approval to conduct the study were obtained from the Palestinian Ministry of Health for the governmental hospitals and from the executive manager of An-Najah National University Teaching Hospital. The data was collected from the medical records with permissions from the Ministry of Health for governmental units and the CEO of the private teaching hospital. No individual names or data are presented in this study. There was no direct contact with the patients. Data was retrieved from the patients’ medical files and from the computerized health information system in some HD centers.

### Data collection

The HD centers included in the study were: Alia hospital (Hebron), Palestinian medical complex (Ramallah), Beit Jala hospital (Bethlehem), Dr. Thabit Thabit hospital (Tulkarm), Dr. Darweesh Nazzal hospital (Qalqelia), Dr. Khalil Sulaiman hospital (Jenin), Jericho hospital (Jericho), Al-Shaheed Yaser Arafat hospital (Salfeet) and An-Najah National University Teaching hospital (Nablus). The overall number of patients undergoing HD in each center was recorded and those who have HBV and HCV in each center were recorded as well.

The patients were included in the study only if they were attending HD center and undergoing HD on a regular schedule for more than one month. Patients who underwent HD for less than one month were excluded.

### Statistical analysis

The data was collected, entered, summarized, tabulated and analyzed using the Statistical Package for Social Sciences (SPSS) software version 16 [9]. Descriptive statistics was performed on the collected data and on each center separately.

## Results

This study focuses on determining the prevalence of both HBV and HCV among HD patients in the West Bank.

### Patients’ distribution among hemodialysis centers

The study was conducted on 868 HD patients attending nine HD centers in the West Bank. Table 2 below shows the distribution of HD patients by center. The highest number of patients undergoing HD was seen in both Hebron and Nablus while the lowest was found in Jericho. As shown in the table, the highest number of patients with HBV was found in Beit Jala (11 cases) while for HCV, the highest number was found in Jenin (18 cases). However, Jericho and Qalqelia had the lowest (zero) number of HBV. Regarding HCV, the lowest number of patients was seen in Jericho (one case).

**Table 2:** Patients distributions and numbers with HBV and HCV by hemodialysis centers in the West Bank

Hemodialysis center	N° of patients	n° with HBV^a^	n° with HCV ^a^
Hebron	177	9	13
Ramallah	135	4	5
Nablus	174	2	5
Beit Jala	93	11	8
Tulkarm	71	2	4
Qalqelia	44	0	7
Jenin	117	4	18
Jericho	25	0	1
Salfeet	32	1	3
Total	868	33	64

^a^ *HBV* hepatitis B virus, *HCV* hepatitis C virus

### Prevalence of HBV and HCV by hemodialysis centers

**Fig. 1:**
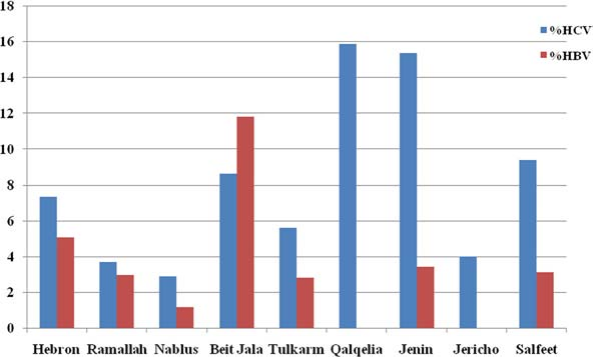
Prevalence of HBV and HCV by hemodialysis center in the West Bank. HBV: Hepatitis B virus; HCV: Hepatitis C virus

Figure 1 below shows the prevalence of both HBV and HCV in each HD center in the West Bank. The overall prevalence of HBV among HD patients in the West Bank was 3.8 % with a range from 0.0 % in Jericho and Qalqelia to 11.8 % in Bethlehem. Regarding HCV, the overall prevalence among HD patients in the West Bank was 7.4 % with a range from 2.9 % in Nablus to 15.9 % in Qalqelia (see Fig. 1 below).

## Discussion

The highest HBV prevalence in the West Bank was reported in Beit Jala governmental hospital (11.8 %). It is unclear why this high prevalence was reported compared to other West Bank hospitals. However, the result could be attributed to the fact that the hospital was established long time ago and that it provides health care services to different population groups. Moreover, the hospital has a regional referral Oncology department that serves the Palestinian population from South to the Middle of the West Bank and while cancer patients are immune-compromised, they may contribute to a higher level of HBV prevalence in this unit. Furthermore, this prevalence was higher compared to some other countries like USA, Morocco, Iran, but lower than other countries like Pakistan, Spain, Brazil, Turkey and Vietnam (Table 1) [7, 8].

On the other hand, the prevalence of HCV was 2.87 % at An-Najah National University Teaching Hospital in Nablus which was the lowest prevalence of HCV among hemodialysis centers in the West Bank. This prevalence was lower than other countries such as Belgian, Germany, India and Vietnam [7, 8]. This could be explained by that this hospital is a teaching hospital which was recently established and provides well and advanced tertiary services. Therefore, emphasis and attention on preventive measures compared to other hospitals might be more significant in this hospital. However, the prevalence of HCV was 15.90 and 15.38 % in Qalqelia and Jenin districts, respectively. These were the highest prevalences of HCV reported in the West Bank. Indeed, this high prevalence of HCV needs more investigations in order to better understand why the prevalence of HD patients with HCV is so high in these governmental hospitals. However, higher prevalence levels were reported in other countries worldwide such as Greece, Israel, Indonesia, Syria, Tunisia and Lebanon (Table 1).

In Arab countries, the prevalence of chronic HBV among HD patients ranged from 2.0 % in Morocco to 11.8 % in Bahrain [10–12]. Moreover, the prevalence of HCV antibodies among HD patients has been reported to range from 27 % in Lebanon to 48.9 % in Syria [13]. In Jordan, the prevalence of HBV in seven hospitals of the Royal Medical Services was found to be 5.9 % out of the 427 studied subjects [12]. In Casablanca, Morocco a high HCV prevalence (76 %; *N* = 186) was reported compared to relatively small HBV prevalence (2 %; *N* = 186) among chronic hemodialysis patients in the university hospital [11]. In Egypt however, a cross-sectional study was conducted on 2977 individuals. The study determined the prevalence of anti-HCV and HBV surface antigen seropositivity in Damietta Governorate, Egypt. Only 1.1 % were infected with HBV and 9.3 % with HCV [14]. Regarding other parts of the world, in India for example, the prevalence of HBV and HCV were found to be 5.5 and 10.9 %, respectively after starting the HD [15]. Moreover, in spite of the reduction in HBV spread within dialysis centers (for example in our study, the prevalence of HBV in Jericho and Qalqelia governmental units was found to be zero in both districts), some isolated outbreaks of HBV infection continue to be reported among HD patients in developed countries [16].

This study has some limitations. It does not differentiate between patients who developed the hepatitis infection before or after starting the hemodialysis process, therefore, it does not investigate the risk factors of developing HBV and HCV. This would have provided an insight to understand the preventive measures in each hospital. However, this descriptive study could be used as a baseline for future hypothesis generating analytical studies. Furthermore, no other variables were taken into consideration when estimating the prevalence of HBV and HCV among hemodialysis patients such as gender, age, place of residence or other socio-demographic factors. Nevertheless, the study provides a general overview of this health event and urges further future investigations in low and middle income countries to shed the light to this life threatening disorders.

### Recommendations

Further studies should be conducted to evaluate the risk factors of both HBV and HCV among patients undergoing hemodialysis in the West Bank hospitals. These include follow up studies to investigate whether the infection was established before or after starting the hemodialysis to form a more comprehensive opinion on why prevalence values are higher in some Palestinian hospitals compared to other national hospitals. Developing effective strategies to prevent the occurrence of HBV and HCV infections among HD patients is also essential. Investigation of the prevalence and risk factors of both HBV and HCV among hemodialysis patients in the East Jerusalem hospitals is recommended.

## Conclusions

Although relatively low prevalence of both hepatitis B and C viruses was found in a couple of hemodialysis hospitals, some higher prevalence values urge for effective prevention control measures and more strict follow up procedures.

## Abbreviations

CKD: chronic kidney disease
ESKD: end stage kidney disease
HBV: hepatitis B virus
HCV: hepatitis C virus
HD: hemodialysis
